# Influencing factors on the quality of recovery after total knee arthroplasty: development of a predictive model

**DOI:** 10.3389/fmed.2024.1427768

**Published:** 2024-08-29

**Authors:** Sen Shan, Qingpeng Shi, Hengyuan Zhang

**Affiliations:** ^1^The Second School of Clinical Medicine, Binzhou Medical University, Yantai, Shandong, China; ^2^Department of Bone and Joint Surgery, Yantai Affiliated Hospital of Binzhou Medical University, Yantai, Shandong, China

**Keywords:** TKA, poor recovery, risk factors, predictive modeling, patient satisfaction

## Abstract

**Introduction:**

Total Knee Arthroplasty (TKA) is a widely performed procedure that significantly benefits patients with severe knee degeneration. However, the recovery outcomes post-surgery can vary significantly among patients. Identifying the factors influencing these outcomes is crucial for improving patient care and satisfaction.

**Methods:**

In this retrospective study, we analyzed 362 TKA cases performed between January 1, 2018, and July 1, 2022. Multivariate logistic regression was employed to identify key predictors of recovery within the first year after surgery.

**Results:**

The analysis revealed that Body Mass Index (BMI), age-adjusted Charlson Comorbidity Index (aCCI), sleep quality, Bone Mineral Density (BMD), and analgesic efficacy were significant predictors of poor recovery (*p* < 0.05). These predictors were used to develop a clinical prediction model, which demonstrated strong predictive ability with an Area Under the Receiver Operating Characteristic (AUC) curve of 0.802. The model was internally validated.

**Discussion:**

The findings suggest that personalized postoperative care and tailored rehabilitation programs based on these predictors could enhance recovery outcomes and increase patient satisfaction following TKA.

## 1 Introduction

TKA is a widely accepted treatment for advanced knee osteoarthritis, aimed at reducing pain, restoring joint function, and enhancing the overall quality of life for patients (1). While TKA procedures are standardized, the outcomes of patient recovery can vary considerably. In the era of personalized medicine, there is a growing focus on creating clinical prediction models that consider various factors to predict recovery outcomes and guide clinical decision-making. Prior studies have identified age, gender, BMI, and socioeconomic status as significant factors influencing recovery after TKA (2–4). However, many current models lack accuracy and relevance because they overlook individual differences and essential factors such as bone health and significant comorbidities (5, 6). The objective of this research is to develop a comprehensive model for predicting postoperative recovery following TKA, incorporating patients' baseline characteristics, backgrounds, and perioperative factors. The hypothesis is that by including a wider array of predictors, the model will offer greater precision and clinical value compared to existing models. Ultimately, the aim is to improve patient outcomes and optimize healthcare resource allocation by providing more accurate and personalized predictions of recovery trajectories post-TKA.

## 2 Materials and methods

### 2.1 Study population and selection criteria

**Figure 1:**
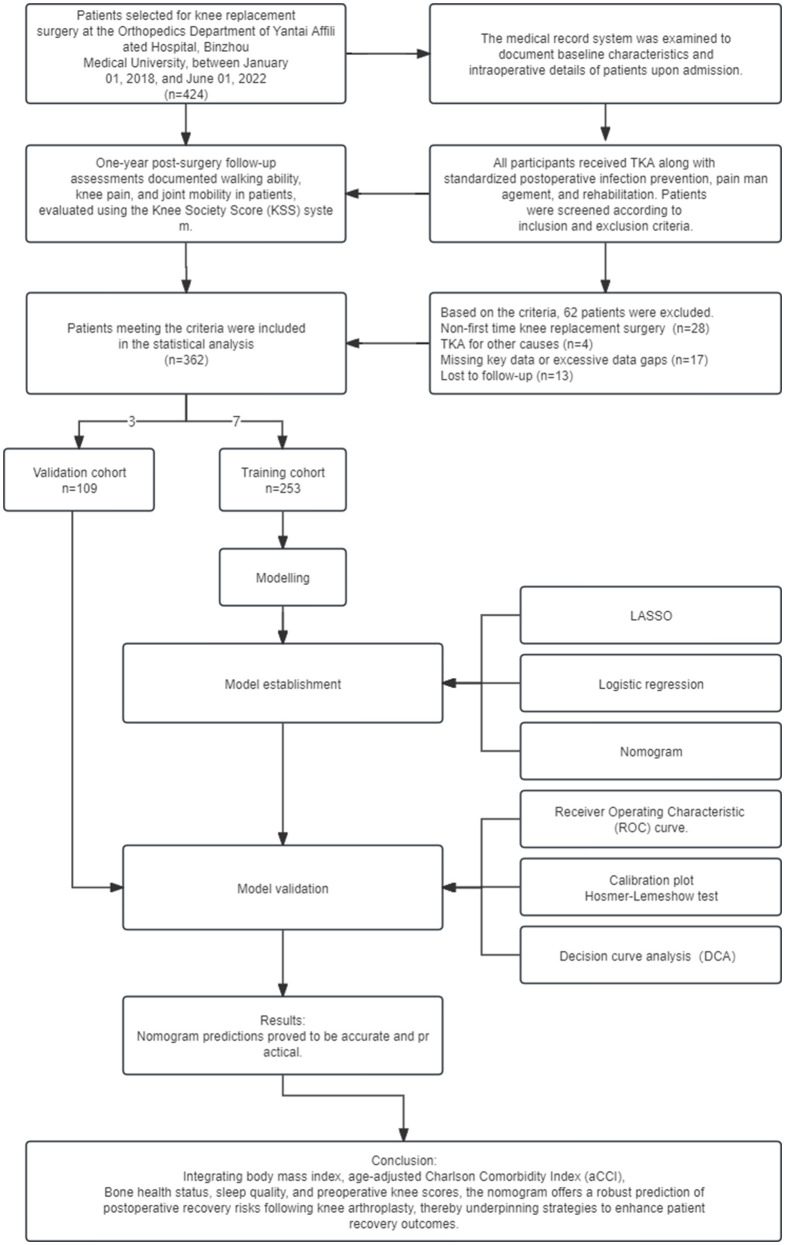
Flowchart for the construction of a clinical predictive model for poor recovery post-TKA.

In this study, 362 patients who underwent TKA surgery at Yantai Affiliated Hospital of Binzhou Medical College between 01/01/2018 and 01/07/2022 were selected. The overall process of this study, including the main steps of research design, data collection, model construction, and validation, is shown in Figure 1. The inclusion criteria were: (1) diagnosed according to the Osteoarthritis Research Society International (OARSI) guidelines for osteoarthritis of the knee published in 2015 (7), focusing on joint space narrowing, imaging evidence of osteophyte formation, and patient-reported symptoms of persistent knee pain and stiffness; (2) underwent the first unilateral TKA procedure; (3) had comprehensive clinical data, including basic information (age, gender, body mass index, and place of residence), lifestyle habits (smoking, alcohol consumption), medical history (previous surgeries, comorbidities, and medication use), preoperative knee functional status (documented using a KSS score based on knee mobility and pain level), and intra-operative and postoperative management details (duration of tourniquet application, intraoperative blood loss, and type of postoperative analgesia); (4) provided informed consent after being fully informed about the study. Exclusion criteria included a history of knee fractures or surgeries within the year preceding TKA, patients requiring joint replacement due to non-degenerative joint diseases or rheumatoid arthritis, and a history of lumbar spine or brain conditions impacting limb functionality. The latter was evaluated through a review of medical records, patient interviews, and physical examinations by a neurologist or orthopedic specialist to rule out any conditions that could interfere with the study outcomes. Additionally, patients with incomplete data significantly impacting analysis and outcomes, and those lost to follow-up, were excluded.

### 2.2 Collection of impact factors

All surgical operations and related data were conducted by a unified surgical team, thus eliminating the influence of surgical operation variability and indication selection on the results. This study systematically reviewed the literature, conducted clinical observations, and performed theoretical analyses to specifically assess patient variables, including age, gender, height, weight, history of previous diseases, smoking and drinking habits, bone mineral density, quality of sleep, place of residence, preoperative knee function, duration of intraoperative tourniquet use, and intraoperative bleeding. To ensure the accuracy and completeness of the study results, we excluded patients who lacked critical data. During preoperative and postoperative evaluations, we simplified and optimized the variables by referring to criteria from relevant literature. BMI was calculated using height and weight, and patients' age and comorbidities were assessed using the modified Charlson comorbidity index (aCCI), with higher scores indicating poorer health status (8). All patients underwent preoperative bone mineral density testing and were classified as having normal (T-score > −1SD), reduced bone mineral density (T-score between −1SD and −2.5SD), or osteoporosis (T-score < −2.5SD) skeletal health according to the World Health Organization (WHO) guidelines for grading osteoporosis. To accurately assess sleep quality (9), we applied the Pittsburgh Sleep Quality Index (PSQI) to all patients, categorizing them into two groups: good sleep and sleep disorders, based on a 5-point scale according to the Buysse study (10). Preoperative knee function was assessed using the Knee Society Score (KSS) (11), providing a comprehensive picture of knee function, pain level, stability, and range of motion. Recent literature suggests that age and diabetes may independently affect postoperative recovery (12, 13). Consequently, this study meticulously integrated these two factors along with other collected variables into the analysis to attain a more nuanced understanding and comprehensive evaluation of their specific impacts on postoperative outcomes. Through this comprehensive assessment, we aim to provide scientific guidance for TKA postoperative recovery and ultimately enhance patients' quality of life.

### 2.3 Study design

In order to construct and validate a predictive model for the quality of recovery after total knee arthroplasty, a computer-generated random assignment method was used in this study. First, all patients who met the inclusion criteria were numbered. Next, a unique random number was assigned to each patient using computer-generated random numbers. Patients were sorted according to these random numbers and divided into a training set (*n* = 253) and a validation set (*n* = 109) in a 7:3 ratio. Specifically, the top 70% of patients after sorting were assigned to the training set and the remaining 30% to the validation set. This ratio was chosen to ensure the model training had a sufficient sample size while retaining enough patients for validation to assess its generalization ability and stability. The 70% ratio provided enough data for the model to capture patterns and relationships, while the 30% ratio ensured a reliable assessment of the model's performance. This allocation maximized the model's generalization ability without sacrificing accuracy. Using a random number generator for allocation ensured unbiased and randomized patient distribution, thus improving the reliability and applicability of the predictive model across different patient populations (14).

All participants underwent TKA and were provided with standardized postoperative care, including anti-infective, analgesic, and rehabilitation treatments. The recovery process was meticulously monitored through regular follow-up visits scheduled at defined intervals over the first year post-surgery. The assessment of recovery status one year post-surgery was conducted utilizing the KSS. The KSS comprises two principal components: the Function Score, which primarily evaluates the patient's ability to walk, climb stairs, and the necessity for assistive devices; and the Clinical Score, which assesses pain, stability, and the range of motion in the joint. Achieving scores of 60 or above in both the functional and knee categories is indicative of a favorable recovery, whereas scores below 60 in either category are deemed indicative of inadequate recovery. This scoring threshold was established based on insights from previous research (15, 16), with the objective of quantifying recovery outcomes in a rigorous manner. The raw data supporting the findings of this study are available in the Supplementary File 1.

### 2.4 Statistical methods

This study employed SPSS 26.0 (IBM SPSS Statistics 26.0) and R version 4.3.1 for meticulous data processing and the development of the predictive model. Initial analysis included assessing normality with the Shapiro-Wilk test, with normally distributed variables (e.g., age, BMI) reported as mean ± SD, and others (e.g., length of history, aCCI) presented as median and IQR. Variable comparisons utilized *t*-tests or Mann-Whitney U-tests based on distribution, with categorical variables analyzed via chi-square tests. LASSO regression was strategically applied to select significant predictors for logistic regression analysis, resulting in a robust predictive model visualized with a column line graph. In validating the predictive model, calibration curves were utilized to examine the consistency of the model's predictions with observed outcomes, ensuring its reliability. ROC curves and their corresponding area under the curve (AUC) values were employed to assess the model's discriminative ability, identifying its capability to distinguish between different outcome categories effectively. Decision curve analysis (DCA) further evaluated the model's clinical utility, demonstrating its benefit in guiding clinical decision-making. Throughout this study, a significance level of *P* < 0.05 was adopted, denoting that findings with a probability of occurring by chance less than 5% were considered statistically significant.

## 3 Results

In this study, we meticulously compared the baseline demographic and clinical characteristics between the training set (*n* = 253) and the test set (*n* = 109) as shown in Table 1. Statistical analysis, employing independent *t*-tests for continuous variables and chi-square tests for categorical variables, revealed no significant differences in key metrics such as age, gender, BMI, and prevalent comorbidities between the two cohorts, with *p*-values ranging from 0.095 to 0.945. This thorough comparison ensures consistency and comparability among participants, affirming the absence of significant disparities. The robustness of the predictive model's applicability across different patient groups is highlighted, thereby enhancing its reliability.

**Table 1:** Patient demographics and baseline characteristics.

**Characteristic**		**Cohort**		
		**Training set**, ***N*** = **253**^a^	**Test set**, ***N*** = **109**^a^	***p*** **-value** ^b^
Sexes	Women	187 (73.9%)	80 (73.4%)	0.918
	Male	66 (26.1%)	29 (26.6%)	
Current address	Municipalities	209 (82.6%)	94 (86.2%)	0.391
	Villagers	44 (17.4%)	15 (13.8%)	
Hypertensive	No	112 (44.3%)	47 (43.1%)	0.84
	Yes	141 (55.7%)	62 (56.9%)	
Cigarette smoking	No	226 (89.3%)	96 (88.1%)	0.727
	Yes	27 (10.7%)	13 (11.9%)	
Drinking wine	No	223 (88.1%)	94 (86.2%)	0.615
	Yes	30 (11.9%)	15 (13.8%)	
BMD^d^	Normal	148 (58.5%)	70 (64.2%)	0.359
	Osteopenia	60 (23.7%)	26 (23.9%)	
	Osteoporosis	45 (17.8%)	13 (11.9%)	
Diabetes	No	178 (70.4%)	72 (66.1%)	0.417
	Yes	75 (29.6%)	37 (33.9%)	
Intraoperative hemorrhage	< 200 ml	194 (76.7%)	80 (73.4%)	0.504
	≥200 ml	59 (23.3%)	29 (26.6%)	
Tourniquet application time	< 90 min	204 (80.6%)	86 (78.9%)	0.705
	≥90 min	49 (19.4%)	23 (21.1%)	
Sleep quality	Sleep well	142 (56.1%)	52 (47.7%)	0.141
	Sleep disorder	111 (43.9%)	57 (52.3%)	
Postoperative analgesia	Intravenous self-controlled analgesia	78 (30.8%)	34 (31.2%)	0.945
	Intermittent intravenous administration	175 (69.2%)	75 (68.8%)	
Age	Mean ± SD	67.8 ± 5.6	66.8 ± 5.6	0.095
BMI^c^	Mean ± SD	27.1 ± 3.4	26.6 ± 3.4	0.222
Duration of illness (years)	Median (IQR)	6.0 (3.0, 10.0)	10.0 (3.0, 10.0)	0.26
aCCI^e^	Median (IQR)	4.00 (3.00, 5.00)	3.00(3.00, 4.00)	0.418
Preoperative KSS score	Median (IQR)	88 (69, 107)	88 (59, 99)	0.3

^a^n (%).

^b^Pearson's Chi-squared test; Welch Two Sample t-test; Wilcoxon rank sum test.

^c^BMI, Body Mass Index.

^d^BMD, Bone Mineral Density.

^e^aCCI, age-adjusted Charles comorbidity index.

### 3.1 Screening of potential impact factors

**Figure 2:**
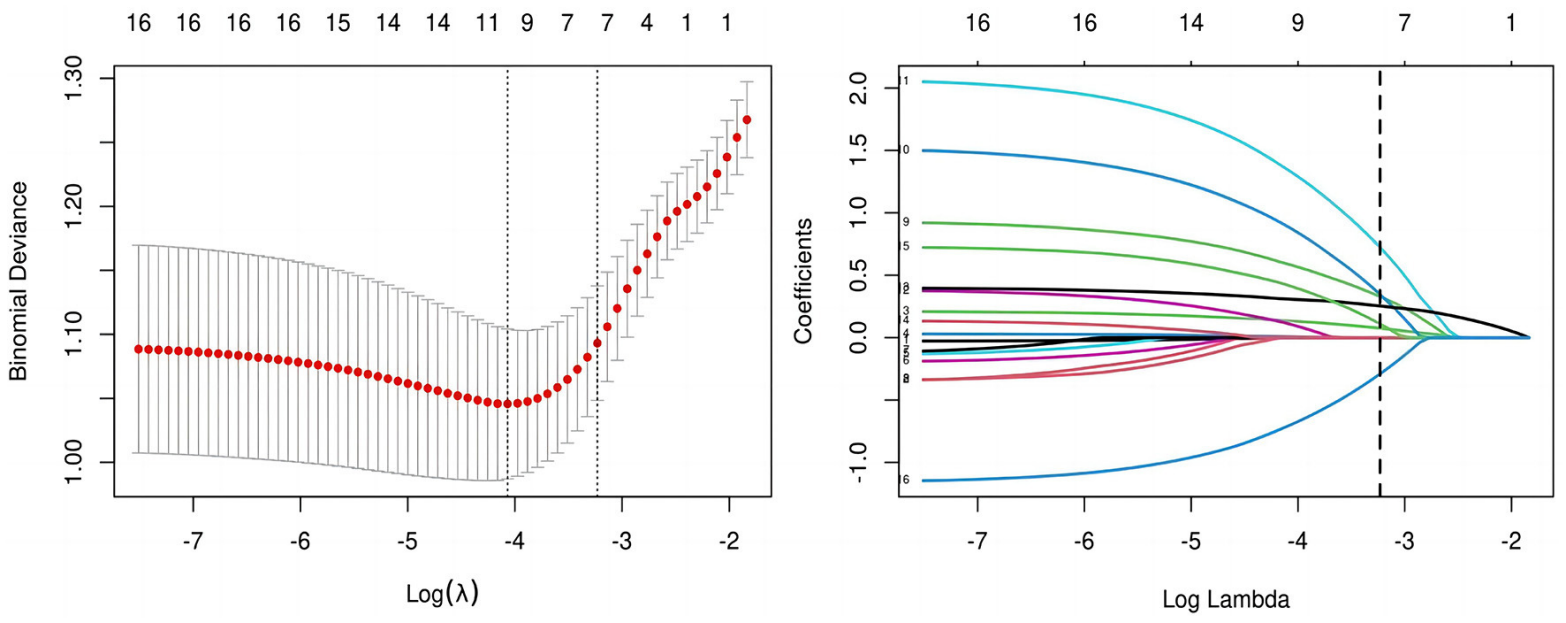
Lasso coefficients and ROC curves for variables.

**Figure 3:**
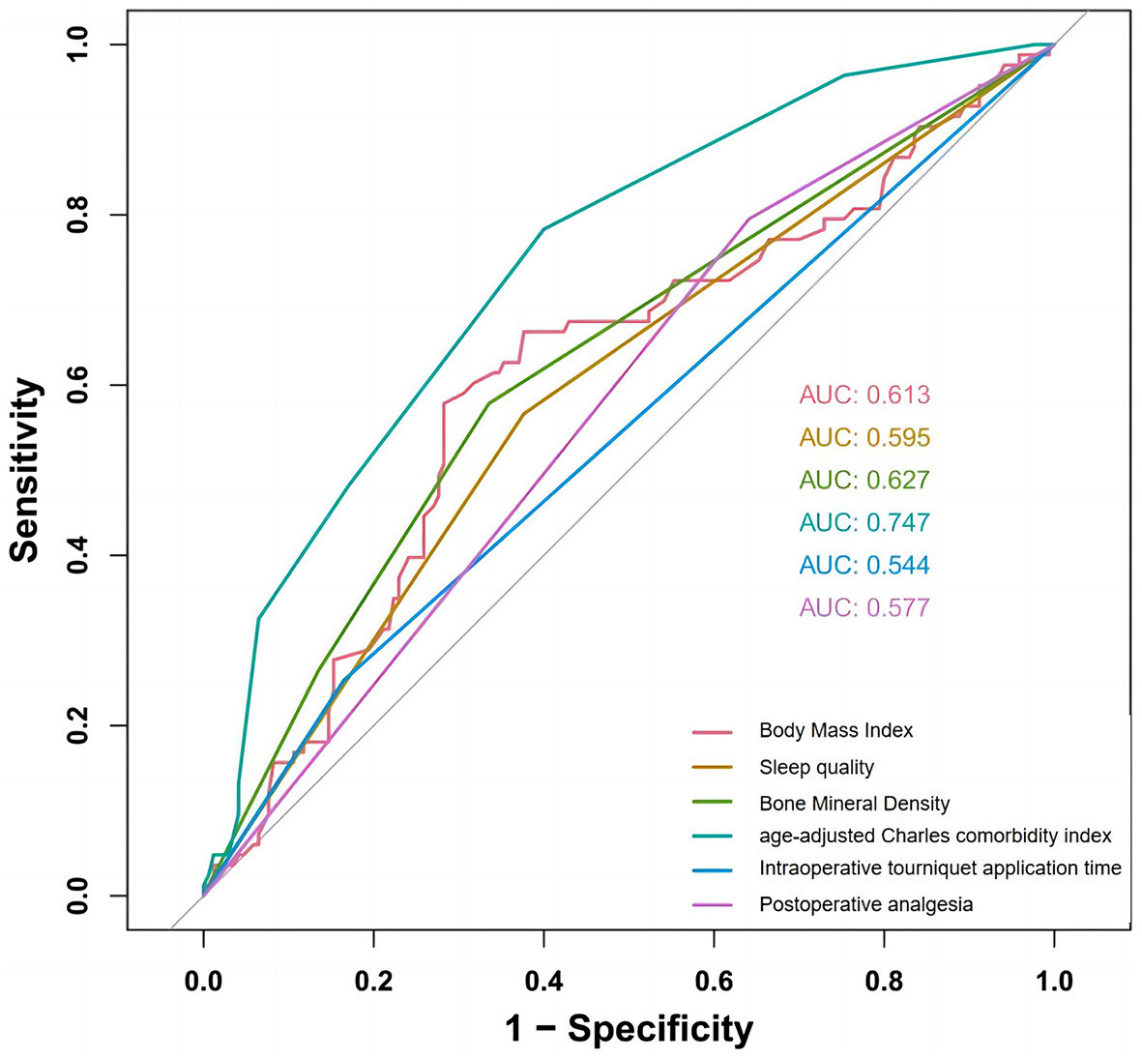
Comparative AUC values from variable-based ROC analysis.

All candidate predictors were incorporated into the original model and then reduced to eight potential predictors using LASSO regression analysis in the training cohort. These predictors included BMI, medical history length, sleep quality, BMD, diabetes, aCCI, tourniquet use duration, and postoperative analgesic method. The coefficients are shown in the table below, and the coefficient profiles are illustrated in Figure 2. A cross-validation error plot for the LASSO regression model is also provided. The most regularized and rational model includes eight variables with cross-validation errors within one standard error of the minimum. To assess the accuracy of the predictive models and the importance of these predictors, ROC curves were generated based on univariate logistic regression predictions. As shown in Figure 3, the ROC analyses of the above variables yielded AUC values greater than 0.5. These curves illustrate the efficacy of the model in discriminating between different recovery outcomes, demonstrating the robustness of the model and the predictive power of the variables.

### 3.2 Analysis and modeling

**Figure 4:**
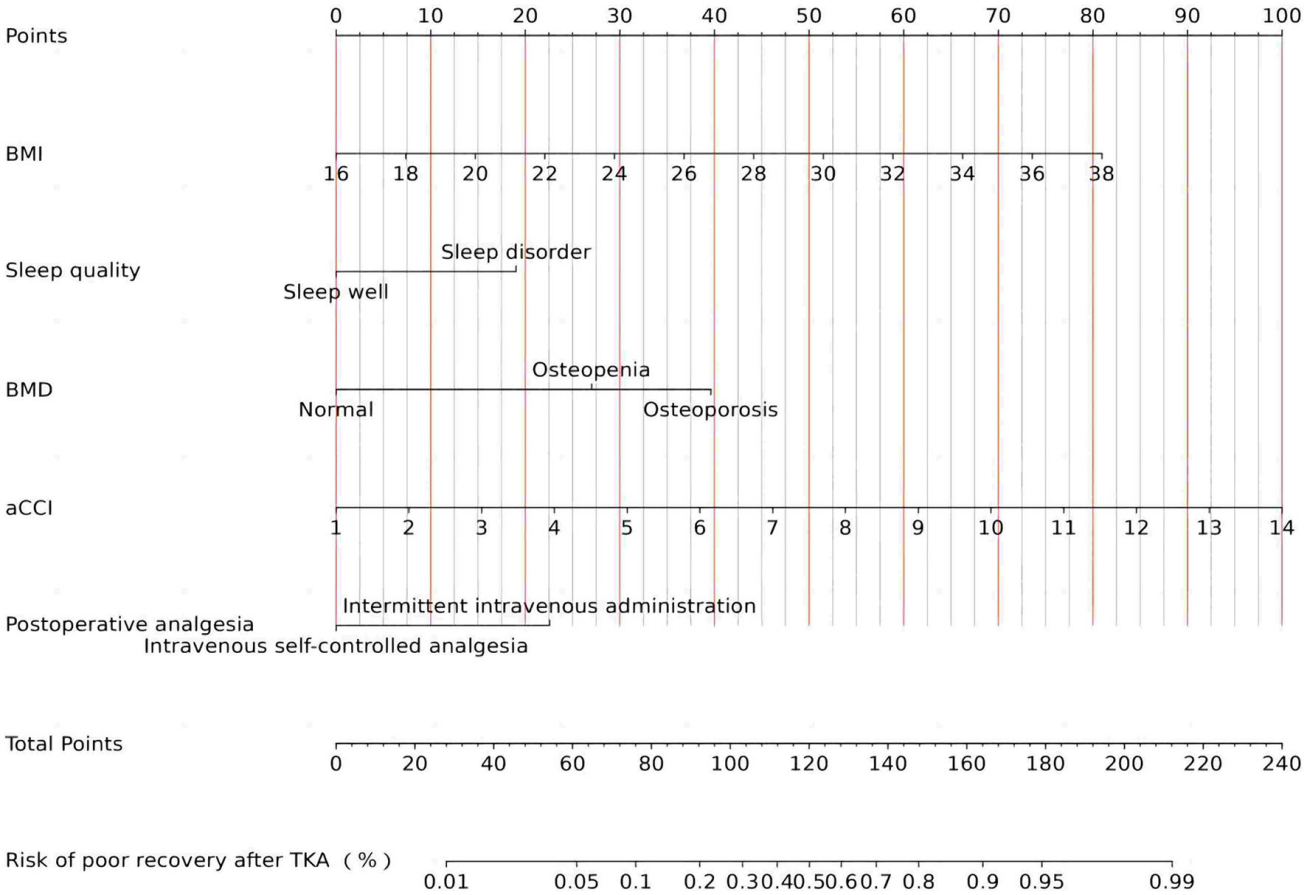
Nomogram for predicting recovery risk post-TKA.

To ascertain whether the eight variables function as independent risk factors for suboptimal recovery following TKA, this study employed multivariate binary logistic regression analysis, carefully adjusting for potential confounders. This robust analysis revealed that factors such as BMI, aCCI, BMD, sleep quality, and postoperative analgesic strategy significantly influence the likelihood of poor recovery outcomes (*p* < 0.05). These pivotal findings are elaborated in Table 2. Additionally, to facilitate practical application, column-line graphical models were constructed based on the identified independent predictors, as illustrated in Figure 4. These models serve as intuitive tools for clinicians and researchers to assess risk and strategize recovery interventions.

**Table 2:** Multiple logistic regression results.

**Characteristic**	**N**	**Event N**	**OR^a^**	**95% CI^a^**	***p*-value**
BMI^**b**^	253	83	1.22	1.11, 1.36	< 0.001
Duration of illness (years)	253	83	1.03	0.98, 1.08	0.281
**Sleep quality**					
Sleep well	142	36	-	-	
Sleep disorder	111	47	2.43	1.26, 4.79	0.009
**BMD** ^**c**^					
Normal	148	35	-	-	
Osteopenia	60	26	4.38	1.96, 10.13	< 0.001
Osteoporosis	45	22	7.58	3.19, 18.88	< 0.001
**diabetes**					
No	178	52	-	-	
Yes	75	31	1.44	0.71, 2.93	0.309
aCCI^d^	253	83	1.44	1.21, 1.75	< 0.001
**Tourniquet application time**					
< 90 min	204	62	-	-	
≥90 min	49	21	2.11	0.96, 4.63	0.061
**Postoperative analgesia**					
Intravenous self-controlled analgesia	78	17	-	-	
Intermittent intravenous administration	175	66	3.21	1.54, 7.09	0.003

^a^OR, Odds Ratio; CI, Confidence Interval.

^b^BMI, Body Mass Index.

^c^BMD, Bone Mineral Density.

^d^aCCI, age-adjusted Charles comorbidity index.

### 3.3 Model validation

**Figure 5:**
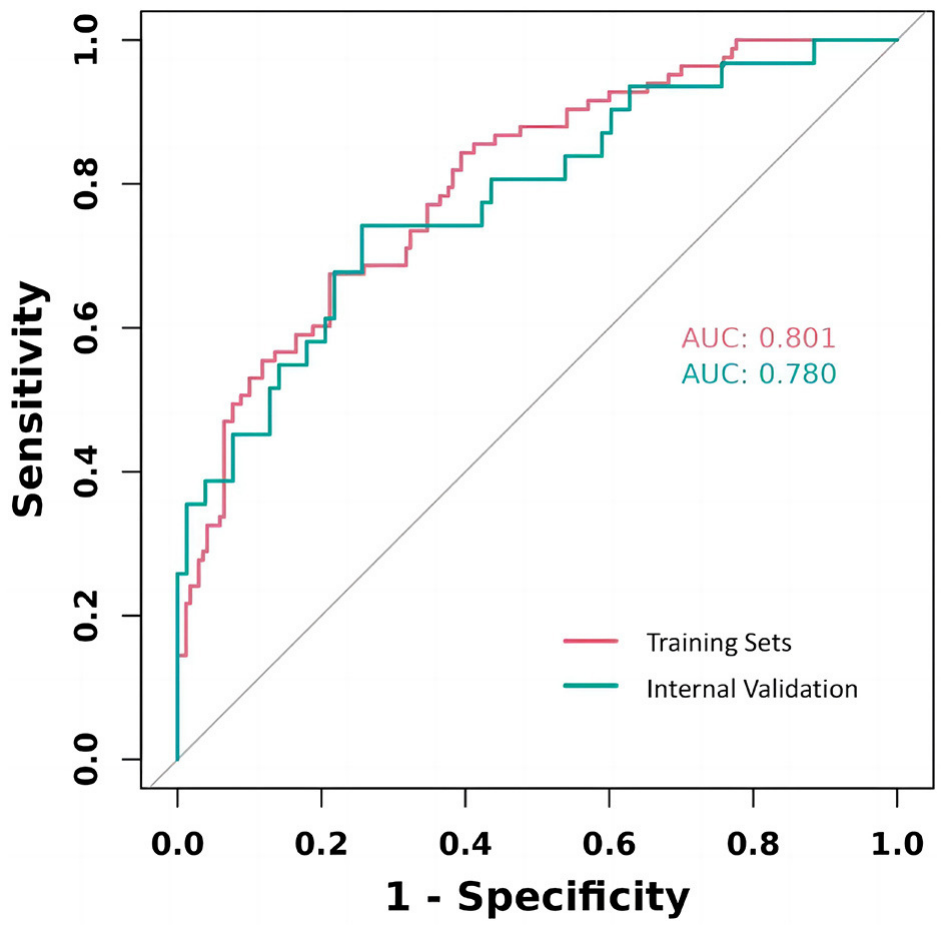
ROC curves for TKA prognostic model training and validation.

The study rigorously assessed the prediction model's performance by calculating the area under the ROC for each cohort, revealing a strong validation performance (AUC = 0.802). This AUC value signifies a high degree of model accuracy in distinguishing between outcomes, underscoring the model's effectiveness. The similarity of ROC curves across training and validation cohorts not only attests to the model's consistency but also its reliability across different patient groups. These results, effectively visualized in Figure 5, underscore the model's potential applicability in clinical settings.

#### 3.3.1 Calibration curve analysis for predictive model accuracy

**Figure 6:**
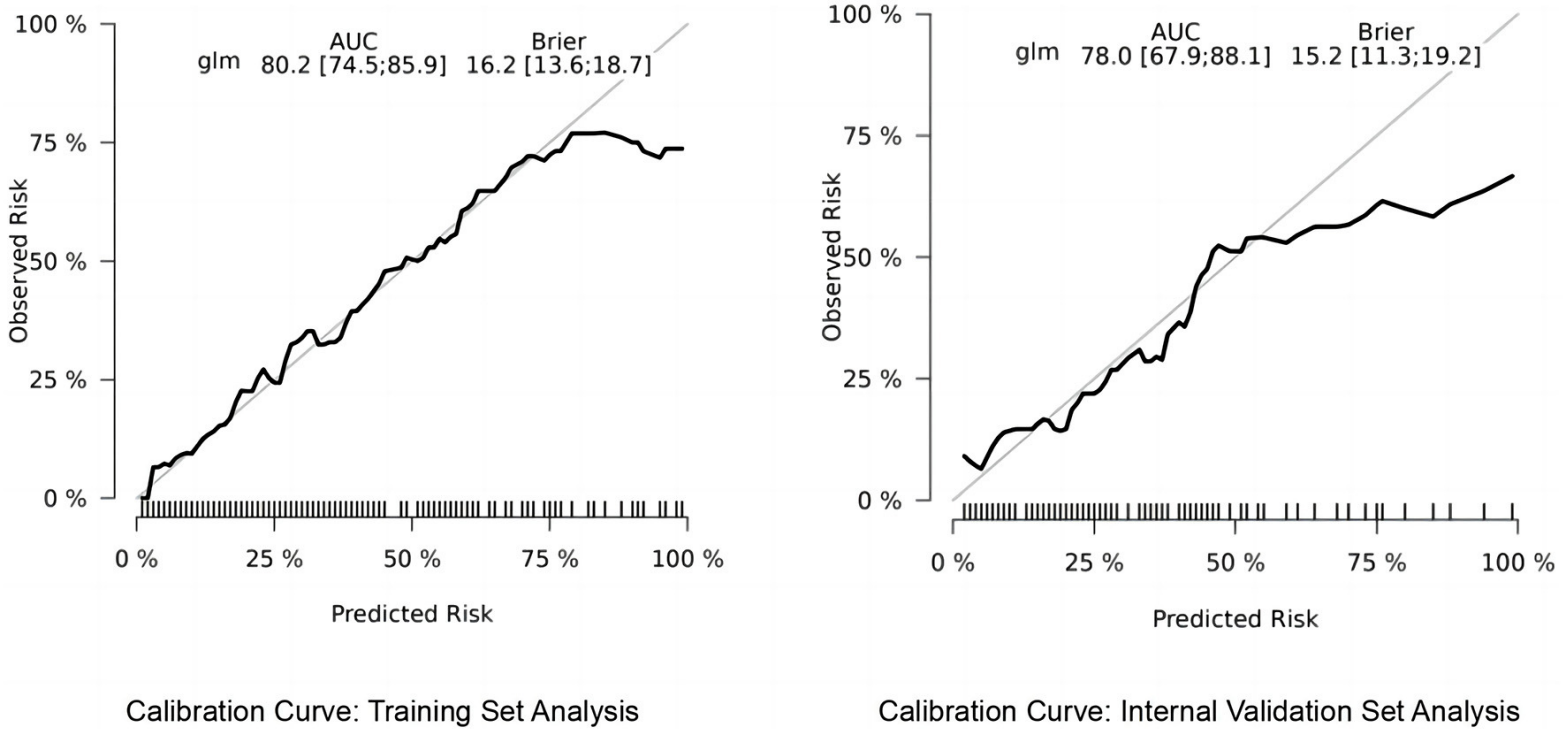
Calibration curves for TKA prognostic model accuracy.

We utilized the risk Regression package in R software to plot calibration curves for both the training and validation sets, examining the correlation between observed and predicted postoperative recovery outcomes. As illustrated in Figure 6, the analysis revealed that the correlation is strong, indicative of the model's robust predictive accuracy. The calibration curves of the original column-line plots for the validation set closely align with ideal curves, demonstrating that the predicted outcomes reliably mirror the actual clinical scenarios.

#### 3.3.2 Decision curve analysis of the predictive model's clinical utility

**Figure 7:**
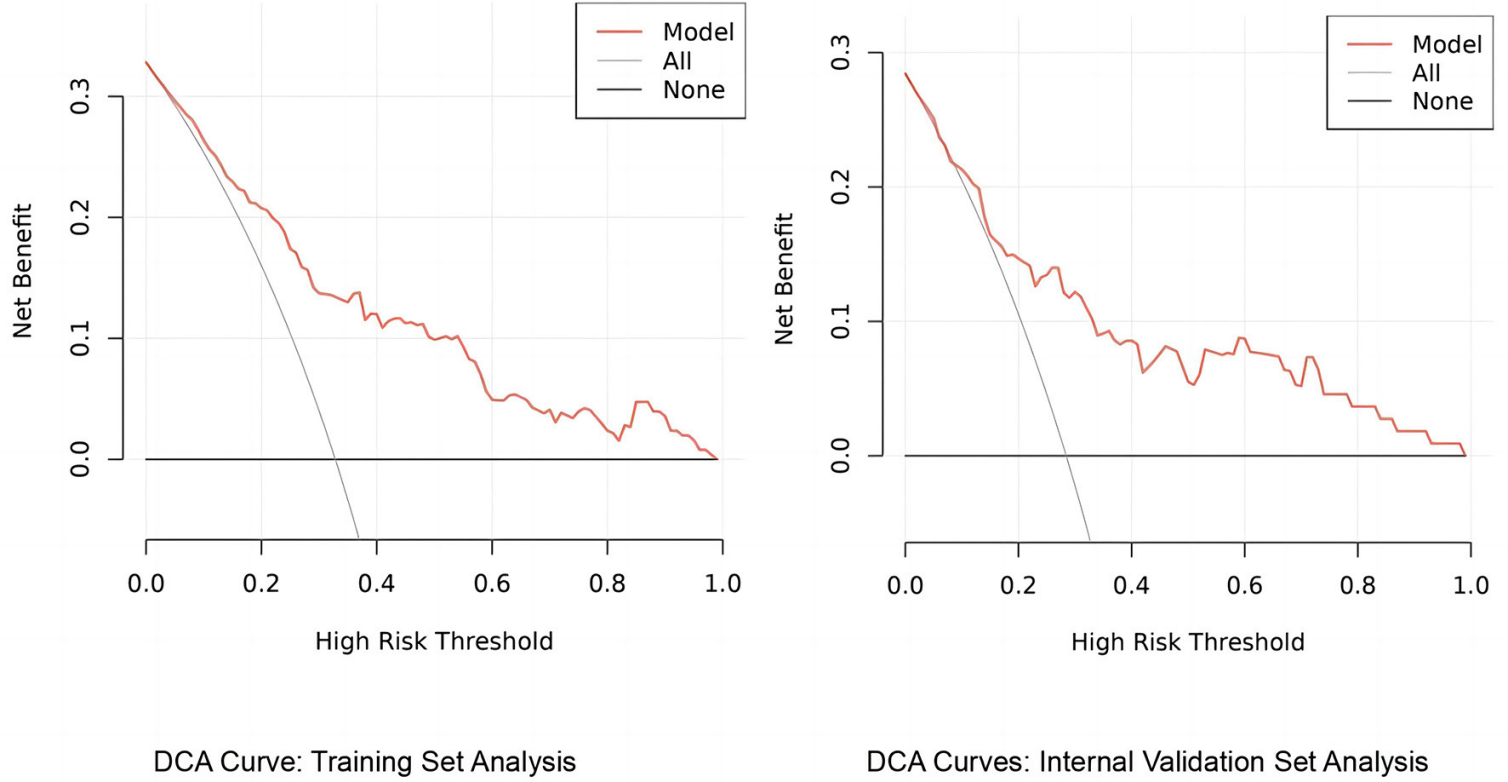
DCA curves for TKA model evaluation.

The column-line graph, generated using the rmda package in R, elucidates the predictive model's utility through decision curve analysis (DCA). This analysis shows that, across a range of clinical decision thresholds, the model consistently offers a higher net benefit than either standard care or opting for no treatment, demonstrating its potential to substantially improve clinical decision-making (see Figure 7). Specifically, the model excels in quantifying the trade-offs between treatment risks and benefits, enabling healthcare providers to tailor decisions to the individual risk profiles of their patients. As a result, our findings advocate for the model's incorporation into clinical settings to optimize treatment plans on a personalized basis, thereby enhancing both the efficacy and efficiency of care.

## 4 Discussion

The study identified key factors affecting postoperative recovery by analyzing 362 patients who underwent TKA and constructed a highly accurate Nomogram model (C-index = 0.802) to provide a scientific basis for preoperative assessment and optimization of postoperative recovery. Our data showed that the KSS scores of patients improved from 81.77 ± 33.58 preoperatively to 139.80 ± 28.38 1 year postoperatively, indicating that TKA significantly improves knee function and reduces pain. Further analysis revealed that factors affecting poor postoperative recovery included BMI, aCCI, BMD, sleep quality, and postoperative analgesic methods. Although our findings align with existing prediction models in many respects, some differences were noted. For example, factors commonly thought to influence postoperative recovery, such as preoperative knee function, diabetes mellitus, hypertension, and age (17–19), did not show a significant effect in this study.

This study found a positive correlation between BMI and quality of recovery after TKA, indicating that patients with higher BMI are at an increased risk of poor postoperative recovery. This finding aligns with previous literature (20). Research has shown that higher BMI is strongly associated with poor postoperative recovery, prolonged hospitalization, increased readmission rates, and risk of complications (21). The slower progression of postoperative knee functional recovery and pain relief in obese patients may result from increased resistance between soft tissues and articular surfaces, as well as heightened stress on surrounding soft tissues due to the prosthesis (15, 22). A growing body of evidence supports that lowering BMI through preoperative interventions can improve recovery after TKA (23, 24). However, it has also been noted that there is not always a direct correlation between high BMI and recovery quality after TKA (25, 26). For instance, a study by Pavlovic showed that BMI or a 5% reduction in preoperative BMI did not significantly affect recovery outcomes at 6 months postoperatively, suggesting that BMI is not the sole influencing factor (27). These differences may stem from variations in study design, sample size, follow-up time, and postoperative outcome assessment metrics. Therefore, future studies with more rigorous designs, larger sample sizes, and long-term follow-up are needed to further explore the specific effects of BMI on postoperative recovery and survival in TKA, considering the potential benefits of individualized and comprehensive interventions. Our study concludes that reducing BMI preoperatively can improve the quality of postoperative recovery, reduce complication rates, and enhance overall outcomes. Applying these findings to clinical practice requires multidisciplinary teamwork to develop and implement individualized preoperative intervention plans to optimize postoperative recovery in TKA patients.

Osteoporosis is prevalent among older adults, reducing bone density and compromising bone structure, thereby making bones more fragile. This condition not only impairs bone healing and slows recovery but also increases the risk of unstable prosthetic fixation and loosening (28). Our data shows that 40% of patients undergoing TKA have varying degrees of bone health issues, with 16% diagnosed with osteoporosis. Among these patients, the rate of poor postoperative recovery was 42.4% for those with bone loss and osteoporosis compared to 24.3% for those without the condition. To validate these observations, we conducted multivariate regression analyses controlling for potential confounders such as age, gender, BMI, and comorbid conditions. The results demonstrated that bone health issues remained a significant independent predictor of suboptimal recovery after TKA, suggesting that improving bone health may be crucial for optimizing postoperative recovery. Notably, a study by Reddy et al. indicated that osteoporosis was prevalent in TKA patients, but treatment rates were low (29). A similar issue was identified in our study during the postoperative follow-up period, where poor treatment compliance due to insufficient awareness of osteoporosis in some patients led to further poor recovery and an increased risk of periprosthetic fracture. This underscores the importance of addressing osteoporosis before and after TKA, highlighting current gaps in recognizing and managing such conditions and their complications. Therefore, to improve the preoperative and postoperative recovery of patients with osteoporosis, the following measures are recommended according to the WHO guidelines for the prevention and management of osteoporosis (30). These measures include the use of calcium and vitamin D supplements and anti-resorptive medications to increase bone density, along with dietary modifications to boost calcium and vitamin D intake. Additionally, the encouragement of moderate weight-bearing and resistance exercise helps build bone and muscle strength. Bone density testing and endocrine assessment are conducted to develop individualized protocols, and raising patient awareness of osteoporosis management through education and psychological support is emphasized. Together, these comprehensive measures can effectively improve preoperative bone health, reduce the incidence of intraoperative and postoperative complications, and enhance the quality of postoperative recovery.

We assessed patients' sleep 1 month before surgery using PSQI scores and found that nearly half of the surgical patients experienced varying degrees of preoperative sleep problems, such as anxiety, insomnia, and sleep disorders due to pain or other causes. To better understand the impact of preoperative sleep quality on postoperative recovery, we further analyzed the data. Our analysis revealed that sleep disorders were an independent risk factor for suboptimal postoperative recovery and that preoperative sleep significantly affected postoperative recovery quality. This finding led us to investigate the specific mechanisms linking preoperative sleep quality and postoperative recovery. We hypothesized that patients with preoperative sleep problems were less tolerant of surgical stress and postoperative pain during hospitalization due to inadequate rest and were more likely to be uncooperative with functional exercises, thus affecting postoperative recovery. Good sleep helps maintain immune function, reduce stress response, improve pain tolerance, enhance psychological status, and support metabolic and cardiovascular health. Conversely, sleep deprivation or poor sleep quality can weaken the immune system, increase the risk of postoperative complications, exacerbate postoperative pain, trigger anxiety and depression, and lead to metabolic and cardiovascular problems that can slow postoperative recovery (31, 32). Several studies have shown that preoperative sleep disorders not only exacerbate postoperative pain and delay wound healing but also affect mental health and attitudes toward recovery (33). Therefore, improving preoperative sleep quality can significantly accelerate the recovery process and improve postoperative satisfaction. To better implement these findings, several measures are recommended for existing patients. These measures include raising patients' awareness of sleep problems through education and psychological support, as well as using cognitive-behavioral therapy, relaxation training, and medication when necessary to improve sleep quality. By implementing these comprehensive interventions, preoperative sleep can be effectively improved, patient tolerance to postoperative stress and pain can be increased, the incidence of postoperative complications can be reduced, and ultimately, the quality of postoperative recovery and patient satisfaction can be enhanced.

In assessing patients' health status, we discarded the traditional method of constructing predictive models by evaluating patients' health with a few simple diseases as variables and instead opted for the more comprehensive aCCI. The aCCI improves the performance and reliability of predictive models by incorporating patients' age and multiple comorbidities to more accurately assess patients' prognosis and health risks. Numerous studies have validated the effectiveness of the aCCI in assessing patient health status (34, 35). The results of the present study showed that an increase in aCCI score was associated with a significant elevation in the risk of poor recovery after TKA, indicating that the overall health status of the patient influences postoperative recovery. This finding supports the clinical application of the aCCI in preoperative risk assessment and emphasizes the importance of integrating the overall health status of the patient in preoperative evaluation (36). Based on the findings of this study, it is recommended that clinicians prioritize the use of the aCCI as an assessment tool for patients' physical status during preoperative evaluation to more accurately predict postoperative recovery. This approach will not only help the healthcare team develop a more personalized treatment plan but also identify high-risk patients preoperatively so that appropriate preventive measures can be taken to reduce complications. Additionally, as a standardized assessment tool, the aCCI is widely applicable and comparable, facilitating the comparison and integration of findings across different medical institutions and providing a solid foundation for further clinical research.

Postoperative analgesia is crucial in the recovery process for patients undergoing knee replacement surgery. In this study, we used a multimodal analgesic strategy (37). NSAIDs were administered for 2 days before the operation, and anti-anxiety medication was given preoperatively depending on the patient's sleep status. During the first 3 postoperative days, two analgesic regimens were used according to the patient's needs: traditional intermittent intravenous administration (IVA) and IV patient-controlled analgesia (PCA). The traditional regimen primarily utilized NSAIDs, while IV PCA included analgesics such as sufentanil and dexmedetomidine. Medication was gradually switched from IV to oral NSAIDs on postoperative day 4. During the perioperative period, we recorded patients' Visual Analog Scale (VAS) scores daily. We observed that many patients receiving traditional intermittent intravenous analgesic administration experienced significant pain peaks and troughs on postoperative days 1–2, necessitating supplemental analgesic administration. This did not occur in patients who received IV PCA. In contrast, patients using IV PCA rarely exhibited such fluctuations. Seven days after the operation, the VAS scores of nearly all patients were around 2–3, and there was no significant pain during normal activities, indicating that our analgesic strategy was highly effective. Data analysis revealed that patients using IV PCA showed better functional recovery at 1 year postoperatively compared to those using traditional intermittent intravenous administration. We attribute this to the continuous personalized pain management provided by IV PCA, which allows for more consistent pain control with fewer fluctuations. Additionally, IV PCA enables patients to self-adjust their analgesic dosage according to their needs, reducing the incidence of side effects such as nausea, vomiting, and itching (38). Effective pain control encouraged earlier and more active participation in rehabilitation, optimizing the overall recovery process and significantly improving postoperative functional outcomes (39). Our findings align with existing literature, further validating the superiority of IV PCA in postoperative analgesia management and highlighting its clinical importance in long-term functional recovery (40). This finding provides a crucial basis for clinical practice, supporting the preference for IV PCA in pain management after TKA and reminding physicians to consider its impact on long-term functional recovery when designing postoperative analgesic regimens.

Preoperative knee function, diabetes mellitus, hypertension, and age are often thought to affect postoperative recovery based on previous studies and perceptions (12, 13), but no correlation was found in this study. First, preoperative rehabilitation and improved modern surgical techniques may be the main reasons (41). Many patients received targeted rehabilitation training before surgery, which not only improved the condition of patients with poor preoperative knee function but also enhanced their postoperative recovery. Additionally, advances in modern surgical techniques, including minimally invasive surgery and personalized anesthesia protocols, have significantly reduced surgical trauma, allowing even those with poor preoperative knee function to recover well after surgery (42, 43). Second, the impact of diabetes and hypertension on postoperative recovery has been significantly minimized by modern medical management tools. Diabetic patients usually optimize their health with strict glycemic control and multifactorial interventions such as dietary control and medication prior to surgery. These measures help patients recover better after surgery (44–46). Similarly, patients with hypertension are thoroughly evaluated and managed preoperatively to ensure intraoperative and postoperative blood pressure stabilization, mitigating the negative impact of hypertension on postoperative recovery (30). Furthermore, the effect of age on postoperative recovery becomes insignificant under modern medical conditions. Advances in modern medical technology and multidisciplinary teamwork provide comprehensive treatment and care for elderly patients, ensuring optimal management and support before, during, and after surgery (47). Individual health status, psychological state, and social support systems also largely determine the speed and quality of postoperative recovery. A positive mindset and strong family support in elderly patients significantly contribute to postoperative recovery. Finally, differences in study design and sample characteristics may also explain why traditional variables did not show significant effects in this study. Our study sample was more homogeneous, and both preoperative rehabilitation and surgical management were highly standardized. Specifically, we rigorously screened our study subjects to ensure uniformity in preoperative rehabilitation protocols. Furthermore, our stringent inclusion criteria ensured that all subjects received consistent postoperative care, including standardized pain management, early mobilization, and continuous monitoring for complications. This high level of standardization likely diminished the influence of traditional variables such as preoperative knee function, diabetes, hypertension, and age. Therefore, we believe that the predictors used in this study are more relevant and explanatory within the specific context of modern healthcare. The rigorous design and uniform treatment protocols highlight the importance of contemporary medical practices in shaping patient outcomes, thus providing a more accurate assessment of recovery factors.

## 5 Limitations and future directions

Although this study has made significant progress in exploring the factors that influence recovery after total knee arthroplasty (TKA), there are some limitations. First, the relatively small sample size may limit the external validity of the findings. A small sample size can reduce statistical power and fail to detect certain important effects. Moreover, a limited sample may not accurately represent the broader patient population, making the results applicable only to specific subgroups. Thus, future research should consider increasing the sample size to enhance the reliability and generalizability of the results. Second, this study utilized data from a single center, which may introduce selection bias. Single-center data might not reflect patient conditions and treatment outcomes in diverse healthcare settings, thus limiting the generalizability. To address this issue, future studies should involve multicenter collaborations to gather data from various regions and medical institutions. This approach will not only increase the sample size but also improve the external validity and applicability of the findings. Another limitation was the reliance on self-reported data to evaluate postoperative recovery. The study employed the KSS scoring system, which includes patients' evaluations of their own activities. While subjective assessments are crucial for understanding patient satisfaction with surgery, they can be influenced by factors such as pain tolerance and psychological state, potentially introducing bias. To enhance objectivity, future research should incorporate objective physiological indicators and imaging assessments to provide a comprehensive evaluation of postoperative recovery. In summary, although this study provides valuable insights, its findings should be further validated in larger multicenter studies to ensure broader applicability and reliability.

## 6 Conclusions

This study explored the effects of multiple factors on patient recovery before and after TKA and developed predictive models to help physicians assess patient prognosis and allow patients to self-assess based on their conditions and test results. By implementing this model, clinicians can provide more personalized care, minimizing recovery risks and significantly improving patient satisfaction and quality of life. Although this study has made progress in exploring the factors affecting postoperative recovery after TKA, limitations such as small sample size and single-center data still exist. Future studies should expand the sample size, collaborate in multiple centers, and explore the influence of rehabilitation training and psychological factors in depth to improve the accuracy of the predictive model and the overall quality of patient recovery.

## Data Availability

The original contributions presented in the study are included in the article/Supplementary material, further inquiries can be directed to the corresponding author.

## References

[1] Wylde V Penfold C Rose A Blom AW. Variability in long-term pain and function trajectories after total knee replacement: a cohort study. Orthop Traumatol-Sur. (2019) 105:1345–50. 10.1016/j.otsr.2019.08.014 31594730

[2] Hylkema TH Brouwer S Stewart RE Van Beveren J Rijk PC Brouwer RW. Two-year recovery courses of physical and mental impairments, activity limitations, and participation restrictions after total knee arthroplasty among working-age patients. Disabil Rehabil. (2022) 44:291–300. 10.1080/09638288.2020.1766583 32441539

[3] Shah A Memon M Kay J Wood TJ Tushinski DM Khanna V. Preoperative patient factors affecting length of stay following total knee arthroplasty: a systematic review and meta-analysis. J Arthroplasty. (2019) 34:2124–65.e1. 10.1016/j.arth.2019.04.048 31182407

[4] Pan X Wang J Lin Z Dai W Shi Z. Depression and anxiety are risk factors for postoperative pain-related symptoms and complications in patients undergoing primary total knee arthroplasty in the United States. J Arthroplasty. (2019) 34:2337–46. 10.1016/j.arth.2019.05.035 31229373

[5] Twiggs JG Wakelin EA Fritsch BA Liu DW Solomon MI Parker DA. Clinical and statistical validation of a probabilistic prediction tool of total knee arthroplasty outcome. J Arthroplasty. (2019) 34:2624–31. 10.1016/j.arth.2019.06.007 31262622

[6] Shim J Mclernon DJ Hamilton D Simpson HA Beasley M Macfarlane GJ. Development of a clinical risk score for pain and function following total knee arthroplasty: results from the TRIO study. Rheumatol Adv Pract. (2018) 2:rky021. 10.1093/rap/rky021 30506023 PMC6251482

[7] Kraus VB Blanco FJ Englund M Karsdal MA Lohmander LS. Call for standardized definitions of osteoarthritis and risk stratification for clinical trials and clinical use. Osteoarthritis Cartil. (2015) 23:1233–41. 10.1016/j.joca.2015.03.036 25865392 PMC4516635

[8] Charlson ME Pompei P Ales KL MacKenzie CR A. new method of classifying prognostic comorbidity in longitudinal studies: development and validation. J Chronic Dis. (1987) 40:373–83. 10.1016/0021-9681(87)90171-8 3558716

[9] Kanis JA McCloskey EV Johansson H Oden A Melton LJ Khaltaev N. Reference standard for the description of osteoporosis. Bone. (2008) 42:467–75. 10.1016/j.bone.2007.11.001 18180210

[10] Buysse DJ Reynolds CF Monk TH Berman SR Kupfer DJ. The Pittsburgh sleep quality index: A new instrument for psychiatric practice and research. Psychiatry Res. (1989) 28:193–213. 10.1016/0165-1781(89)90047-4 2748771

[11] Liow RYL Walker K Wajid MA Bedi G Lennox CME. The reliability of the American Knee Society Score. Acta Orthop Scand. (2000) 71:603–8. 10.1080/000164700317362244 11145388

[12] Sweerts L Hoogeboom TJ Van Wessel T Van Der Wees PJ Van De Groes SAW. Development of prediction models for complications after primary total hip and knee arthroplasty: a single-centre retrospective cohort study in the Netherlands. BMJ Open. (2022) 12:e062065. 10.1136/bmjopen-2022-062065 36002218 PMC9413190

[13] Li H Jiao J Zhang S Tang H Qu X Yue B. Construction and comparison of predictive models for length of stay after total knee arthroplasty: regression model and machine learning analysis based on 1,826 cases in a single Singapore center. J Knee Surg. (2022) 35:007–14. 10.1055/s-0040-1710573 32512596

[14] Gholamy A Kreinovich V Kosheleva O. Why 70/30 or 80/20 relation between training and testing sets: a pedagogical explanation. Int J Intellig Technol Appl Stat. (2018) 11:105–11. 10.6148/IJITAS.201806_11(2).0003

[15] Gupta A Gupta S. Functional success in total knee arthroplasty - does obesity hold the key: a follow-up study. Global J Res Analy. (2022) 11:19–21. 10.36106/gjra/390070231929979

[16] Asif S Choon D. Midterm results of cemented press fit condylar sigma total knee arthroplasty system. J Orthop Surg (Hong Kong). (2005) 13:280–4. 10.1177/230949900501300311 16365492

[17] Namba R Singh A Paxton E Inacio M. Patient factors associated with prolonged postoperative opioid use after total knee arthroplasty. J Arthroplasty. (2018) 8:2449–54. 10.1016/j.arth.2018.03.068 29753617

[18] Amusat N Beaupre L Jhangri G Pohar S Simpson S Warren S. Diabetes that impacts on routine activities predicts slower recovery after total knee arthroplasty: an observational study. J Physiother. (2014) 4:217–23. 10.1016/j.jphys.2014.09.006 25443651

[19] Vissers MM Bussmann J Groot IB Verhaar J Reijman M. Physical functioning four years after total hip and knee arthroplasty. Gait Posture. (2013) 2:310–5. 10.1016/j.gaitpost.2012.12.007 23829981

[20] Abdelmegied WS Sallam A Ahmed MM. Effect of BMI on complications rate, reoperation and functional outcome after total knee arthroplasty, a systematic review of literature. QJM. (2020) 113:hcaa059. 10.1093/qjmed/hcaa059.015

[21] Lai Y-H Cao J Li Z-X Feng W Xu H Zhou Z-K. Effect of body mass index on postoperative mechanical alignment and long-term outcomes after total knee arthroplasty: a retrospective cohort study of 671 knees. Ann Transl Med. (2022) 10:829–829. 10.21037/atm-22-3212 36034999 PMC9403940

[22] Correa-Valderrama A Stangl-Herrera W Echeverry-Vélez A Cantor E Ron-Translateur T Palacio-Villegas JC. Relationship between body mass index and complications during the first 45 days after primary total hip and knee replacement: a single-center study from South America. Clin Orthop Surg. (2019) 11:159. 10.4055/cios.2019.11.2.159 31156766 PMC6526130

[23] Kostic AM Leifer VP Gong Y Robinson MK Collins JE Neogi T. Cost-effectiveness of surgical weight-loss interventions for patients with knee osteoarthritis and class iii obesity. Arthritis Care Res (Hoboken). (2023) 75:491–500. 10.1002/acr.24967 35657632 PMC9827536

[24] Salis Z Sainsbury A. Association between change in body mass index and knee and hip replacements: a survival analysis of seven to ten years using multicohort data. Arthritis Care Res. (2023) 75:1340–50. 10.1002/acr.25021 36106942 PMC10953021

[25] Goutham B Vanga MR Sidda S. The effect of body mass index on functional outcome in total knee replacement. Int J Orthop Sci. (2021) 7:548–552. 10.22271/ortho.2021.v7.i1i.2540

[26] Reddy SN Goutham B Venkatesh Y. Role of body mass index on total knee replacement rehabilitation and outcome. Nat J Clin Orthop. (2020) 4:41–43. 10.33545/orthor.2020.v4.i4a.247

[27] Pavlovic N Harris IA Boland R Brady B Genel F Naylor J. The effect of body mass index and preoperative weight loss in people with obesity on postoperative outcomes to 6 months following total hip or knee arthroplasty: a retrospective study. Arthroplasty. (2023) 5:48. 10.1186/s42836-023-00203-5 37777817 PMC10544191

[28] Mohammad HR Kennedy JA Mellon SJ Judge A Dodd CA Murray DW. The clinical outcomes of cementless unicompartmental knee replacement in patients with reduced bone mineral density. J Orthop Surg Res. (2020) 15:35. 10.1186/s13018-020-1566-2 32005197 PMC6995049

[29] Ha C-W Park Y-B. Underestimation and undertreatment of osteoporosis in patients awaiting primary total knee arthroplasty. Arch Orthop Trauma Surg. (2020) 140:1109–14. 10.1007/s00402-020-03462-y 32358659

[30] Futier E Lefrant J Guinot P Godet T Lorne E Cuvillon P. Effect of individualized vs standard blood pressure management strategies on postoperative organ dysfunction among high-risk patients undergoing major surgery: a randomized clinical trial. JAMA (2017) 318:14172. 10.1001/jama.2017.14172 28973220 PMC5710560

[31] Rampes S Ma K Divecha YA Alam A Ma D. Postoperative sleep disorders and their potential impacts on surgical outcomes. J Biomed Res. (2020) 34:271. 10.7555/JBR.33.20190054 32519977 PMC7386412

[32] Su X Wang D-X. Improve postoperative sleep: what can we do? Curr Opin Anaesthesiol. (2018) 31:83–8. 10.1097/ACO.0000000000000538 29120927 PMC5768217

[33] Van Meirhaeghe JP Salmon LJ O'Sullivan MD Gooden BR Lyons MC Pinczewski LA. Improvement in sleep patterns after hip and knee arthroplasty: a prospective study in 780 patients. J Arthroplasty. (2021) 36:442–8. 10.1016/j.arth.2020.08.056 32948424

[34] Jianda X Homma Y Jinnai Y Baba T Zhuang X Watari T. Relationship between Charlson comorbidity index, early recovery and 2-year mortality in elderly patients undergoing surgical treatment of inter-trochanteric fractures: a retrospective analysis. Sci Rep. (2021) 11:17195. 10.1038/s41598-021-96765-y 34433884 PMC8387360

[35] Shinonara K Ugawa R Arataki S Nakahara S Takeuchi K. Charlson comorbidity index is predictive of postoperative clinical outcome after single-level posterior lumbar interbody fusion surgery. J Orthop Surg Res. (2021) 16:235. 10.1186/s13018-021-02377-7 33785033 PMC8008557

[36] Abdullah M Al-Salamah SM. Impact of comorbidity on outcome among acute non-traumatic surgical patients. Evaluation of Charlson comorbidity index. Saudi Med J. (2009) 30:228–33. 19198711

[37] Lachiewicz PF. Perioperative pain management in orthopedic surgery. Orthopedics. (2013) 36:6. 10.3928/01477447-20130122-50 23379569

[38] Lavand'homme PM Kehlet H Rawal N Joshi GP PROSPECT PROSPECT Working Group of the European Society of Regional Anaesthesia and Pain Therapy (ESRA). Pain management after total knee arthroplasty: PROcedure SPEcific Postoperative Pain ManagemenT recommendations. Eur J Anaesthesiol. (2022) 39:743–757. 10.1097/EJA.0000000000001691 35852550 PMC9891300

[39] Wylde V Dennis J Gooberman-Hill R Beswick AD. Effectiveness of postdischarge interventions for reducing the severity of chronic pain after total knee replacement: systematic review of randomised controlled trials. BMJ Open. (2018) 8:e020368. 10.1136/bmjopen-2017-020368 29490967 PMC5855247

[40] Viscusi E. Patient-controlled drug delivery for acute postoperative pain management: a review of current and emerging technologies. Region Anesthes Pain Med. (2007) 33:146–58. 10.1136/rapm-00115550-200803000-00010 18299096

[41] Vedoya SP Sel HD. Total knee arthroplasty and extra-articular deformity: deformity correction with intra-articular bone resections. 10 years follow up. J Orthopaed. (2021) 23:219–24. 10.1016/j.jor.2021.01.007 33642818 PMC7887331

[42] Aglietti P Baldini A Giron F Sensi L. Minimally invasive total knee arthroplasty: is it for everybody? HSS J. (2006) 2:22–6. 10.1007/s11420-005-0127-x 18751842 PMC2504119

[43] Krych A Horlocker T Hebl J Pagnano M. Contemporary pain management strategies for minimally invasive total knee arthroplasty. Instr Course Lect. (2010) 59:99–109. 20415373

[44] Nwadiugwu MC. Inflammatory activities in type 2 diabetes patients with co-morbid angiopathies and exploring beneficial interventions: a systematic review. Front Public Health. (2021) 8:600427. 10.3389/fpubh.2020.600427 33569370 PMC7868423

[45] Ho C-J Chen Y-T Wu H-L Huang H-T Lin S-Y. The effects of a patient-specific integrated education program on pain, perioperative anxiety, and functional recovery following total knee replacement. JPM. (2022) 12:719. 10.3390/jpm12050719 35629142 PMC9146256

[46] Saiki Y Ojima T Kabata T Kubo N Hayashi S Tsuchiya H. Gradual exacerbation of knee flexion angle after total knee arthroplasty in patients with diabetes mellitus. Modern Rheumatol. (2021) 31:1215–20. 10.1080/14397595.2020.1868688 33428492

[47] Gupta S Rane A. Enhanced recovery after surgery: perspective in elder women. J Midlife Health. (2021) 12:93–8. 10.4103/jmh.jmh_89_21 34526741 PMC8409712

